# Small Non-Coding RNAs as New Biomarkers to Evaluate the Quality of the Embryo in the IVF Process

**DOI:** 10.3390/biom12111687

**Published:** 2022-11-14

**Authors:** Silvia Toporcerová, Ivana Špaková, Katarína Šoltys, Zuzana Klepcová, Marek Kľoc, Júlia Bohošová, Karolína Trachtová, Lucia Peterová, Helena Mičková, Peter Urdzík, Mária Mareková, Ondřej Slabý, Miroslava Rabajdová

**Affiliations:** 1 Department of Gynaecology and Obstetrics, Faculty of Medicine, Pavol Jozef Šafárik University in Košice, 040 11 Košice, Slovakia; 2 Gyncare a.s., 040 13 Košice, Slovakia; 3 Department of Medical and Clinical Biochemistry, Faculty of Medicine, Pavol Jozef Šafárik University in Košice, 040 11 Košice, Slovakia; 4 Faculty of Natural Sciences, Comenius University in Bratislava, 842 15 Bratislava, Slovakia; 5 SAFTRA-BioMAI, Pavol Jozef Šafárik University in Košice, 040 11 Košice, Slovakia; 6 Central European Institute of Technology, Masaryk University, 601 77 Brno, Czech Republic; 7 Department of Medical Biology, Faculty of Medicine, Pavol Jozef Šafárik University in Košice, 040 11 Košice, Slovakia

**Keywords:** miRNA, piRNA, biomarker, IVF, embryo selection, embryonic secretome

## Abstract

The increased interest in assisted reproduction through in vitro fertilization (IVF) leads to an urgent need to identify biomarkers that reliably highly predict the success of pregnancy. Despite advances in diagnostics, treatment, and IVF approaches, the 30% success rate of IVF seems insurmountable. Idiopathic infertility does not have any explanation for IVF failure especially when a patient is treated with a healthy competitive embryo capable of implantation and development. Since appropriate intercellular communication is essential after embryo implantation, the emergence of the investigation of embryonic secretome including short non-coding RNA (sncRNA) molecules is crucial. That’s why biomarker identification, sncRNAs secreted during the IVF process into the blastocyst’s cultivation medium, by the implementation of artificial intelligence opens the door to a better understanding of the bidirectional communication between embryonic cells and the endometrium and so the success of the IVF. This study presents a set of promising new sncRNAs which are revealed to predictively distinguish a high-quality embryo, suitable for an embryo transfer in the IVF process, from a low-quality embryo with 86% accuracy. The identified exact combination of miRNAs/piRNAs as a non-invasively obtained biomarker for quality embryo determination, increasing the likelihood of implantation and the success of pregnancy after an embryo transfer.

## 1. Introduction

**Figure 1:**
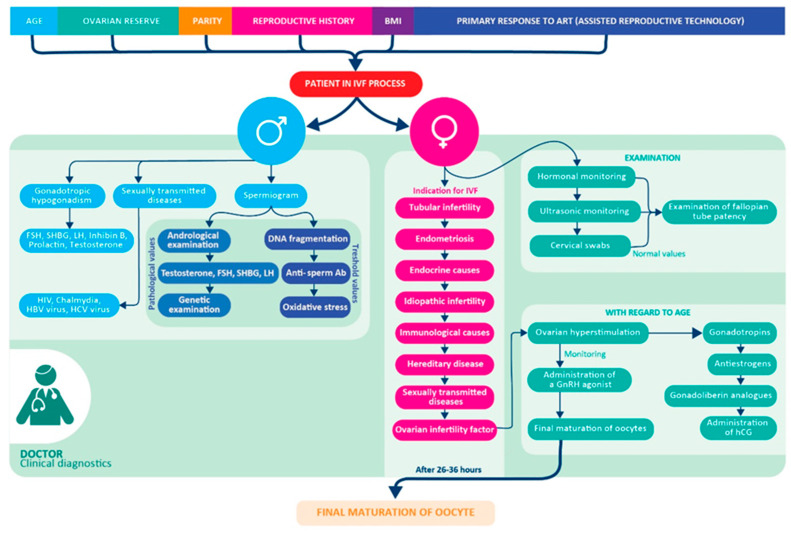
Preclinical and Clinical part of the IVF workflow. Clinical diagnosis of the patient (man and woman) in the IVF process [4].

The inability to conceive after one year of unprotected intercourse, called infertility, exhibits an increasing trend recently [1]. It is estimated to affect 8–12% of couples of reproductive age worldwide [2]. Model predictions for the future suggest that global fertility will continue to diminish [3]. Infertility is becoming more common due to the accumulation of multiple factors, such as gynecological, andrological, hormonal, immunological, hematological, genetic, and age-dependent [4], in both sexes that lead to an inability to conceive. Even if the patient is healthy, hormonally balanced, and seems to be biochemically and genetically compatible with a partner, idiopathic infertility still contributes to one in ten infertility cases [5]. These facts lead to the increased interest in assisted reproduction (AR) through in vitro fertilization (IVF). The overall IVF process is complex, its simplified protocol of the clinical phase is shown in Figure 1.

Although the conditions for embryo cultivation are improving, the overall embryo transfer success rate does not rise above 30% [6]. The success of the IVF process is conditioned also by the receptivity of endometrium, the selection of a competent embryo for transfer, and the determination of the window of implantation (WOI) [7]. The key part of fertilization is cross-talk between the blastocyst (later embryo) the and mother’s endometrium [8] via the secretion of embryo-derived molecules to modulate the uterus compartment and support the blastocyst invasion.

Among the most important signaling pathways of the implantation of the embryo are VEGF (vascular endothelial growth factor) [9]. The family of VEGF and its antagonist sFlt-1 (Soluble Fms-Like Tyrosine Kinase-1) [10], Ang-1/2 (angiopoietin1/2) [11], and endoglin [12] are pivotal for regulation of angiogenesis of mother’s endometrium as well as of embryo. The expression of VEGF, with a significant increase in the mid-luteal phase, suggests the important role of VEGF at the time of embryo implantation [10]. In the first five days of cultivation, the embryonic cells contain among other molecules, also RNAs as well as short non-coding RNAs (sncRNAs), which affect the expression of signaling pathways of implantation [13]. During the IVF process, these embryonic molecules are secreted into a cultivation medium. The competent embryo seems to secrete more of those regulatory molecules for angiogenesis than the incompetent embryo [14].

The ideal embryonic biomarker should be non-invasive, stable, embryo-specific, and easily detectable. The spent blastocyst medium (SBM), a waste cultivation medium, is a non-invasive and easily accessible diagnostic tool [4,15] and is appropriate for the study of sncRNAs as a potential predictive biomarker of embryo quality, suitable for the transfer to the uterus [16].

Human embryos secrete specific microRNAs (miRNAs) and piwi-interacting RNAs (piRNAs) into their extracellular environment, mediating the interaction between the blastocyst and the endometrium, reflecting embryo viability, ploidy as well as implantation potential [13,15,17,18,19,20,21,22] and could be useful in identifying possible causes of implantation failure after an embryo transfer [15,23]. Communication between the embryo and the endometrium is bilateral, therefore, exosomes secreted by the epithelium of the human endometrium transport their contents to the blastocyst, respectively into the adjacent endometrium and thus affect gene expression in the endometrium [18].

The IVF clinics focus on the selection of a single euploid-competent embryo suitable for transfer into the uterine cavity [24]. Only laboratory diagnostics can improve IVF success and the involvement of artificial intelligence appears to be a necessity.

**Figure 2:**
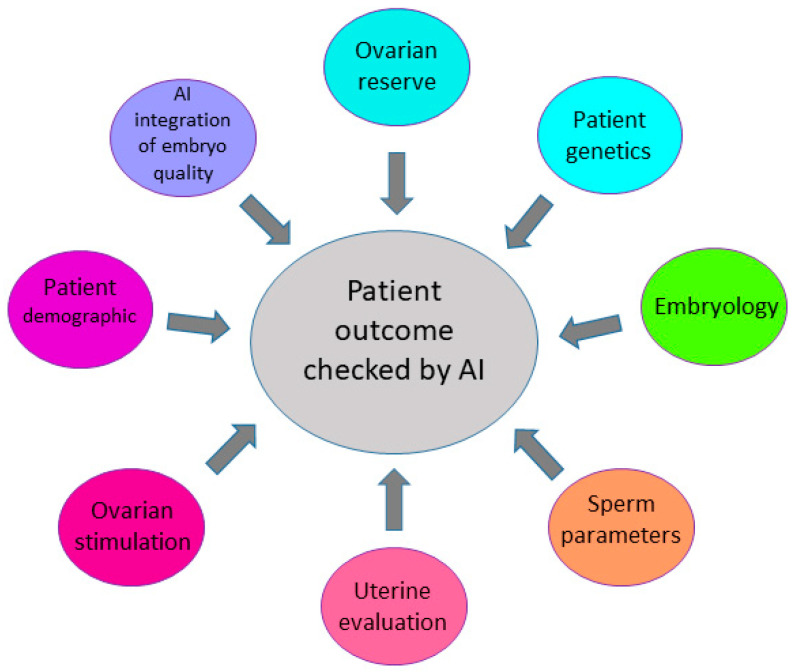
Factors enhancing prediction of the IVF outcome. Several factors, such as ovarian reserve, genetics, sperm parameters, and characterization of embryo quality have an impact on pregnancy outcomes in the IVF process.

The current tool of artificial intelligence (AI) used in clinical practice for embryo evaluation is software analysis using embryo morphology [25] as part of an EmbryoScope (time-lapse monitoring system). However, this method does not provide information about the molecular and biochemical processes of the embryo itself. Machine learning and AI tools can be used to rapidly process and analyze huge amounts of data, create models for clinical decision-making [26] and provide a combination of several significant biomarkers which can describe embryo-patient compatibility [27]. The model of the bioinformatics pipeline of artificial intelligence and machine learning is shown in Figure 2.

Although several miRNAs/piRNAs have been identified as potential biomarkers of successful embryo transfer, the use of artificial intelligence and machine learning approaches on transcriptomics data in this field is still in its beginnings. In the current study, we performed initial small RNA sequencing of the SBM, and data were evaluated using AI to identify promising sncRNAs molecules suitable as noninvasive biomarkers.

## 2. Materials and Methods

### 2.1. Dataset Description

The study has been ongoing since 2018 with the approval of the ethics committee of the Košice governing region on 24 April 2018 (VEGA 1/0873/18), also approved by the ethics committee of Louis Pasteur University Hospital Košice on 21 June 2018 (6026/EK/2018), and continued in the following years based on the approval by the ethics committee of Louis Pasteur University Hospital Košice on 25 April 2019 (4023/EK/2019).

Of the 1113 patients who participated in the study, a group of suitable patients (60 patients) with idiopathic infertility of age from 18 to 37 years old was selected. All participants were not previously on IVF and did not have previous IVF pregnancies. Oocytes collected from these patients went through the process of in vitro fertilization, the embryos were cultured to the blastocyst stage, and the spent blastocyst medium was collected, frozen, and subjected to sncRNA sequencing.

The spent blastocyst medium (10–20 microliters) during the IVF process was collected on the day of the embryo transfer to the uterus (on the 4th or 5th day after fertilization) at the Gyncare Center for Assisted Reproduction in Košice and stored at −70 °C. 

Embryo’s SBM samples (n = 60) were collected after embryo cultivation and control samples of cultivation medium with additive components needed for embryo cultivation as a qualitative and quantitative background (n = 3) were used for sequencing (n = 60 + 3 = 63). Of the total number of sequenced SBM samples (n = 63), 53% (n = 32) were successfully implanted, and 47% (n = 28) were non-implanted. The success and the failure of IVF were determined by the result of the hCG level as well as USG tested on day 10 after the embryo transfer. A level of hCG above 10 IU/L and positive USG monitoring was considered a positive pregnancy.

The studied 60 SBM samples were divided into the exploratory phase the study (n = 48) and the validation phase of the study (n = 12).

### 2.2. SncRNAs Sequencing

Using the method of massive-parallel sequencing of small non-coding RNA molecules (miRNAs and piRNAs) on the Illumina sequencing platform was performed. Based on specific Explanatory Data Analysis and Machine learning methods with different models, the number of predictive biomarkers, and specific prognostic-predictive molecules were selected.

Total RNA enriched for small RNAs were extracted from collected SBM samples using miRNeasy Serum/Plasma Kit (Qiagen, Hilden, Germany) according to the manufacturer’s protocol. The cDNA libraries for next-generation sequencing were prepared using the QiaSeq miRNA Library Kit (Qiagen). The concentrations of cDNA libraries were assessed by fluorometry (Qubit 2.0, Thermofisher Scientific, Waltham, MA, USA) and the equimolar amounts of each library were pooled at a final concentration of 2 nmol/L cDNA. Pooled cDNA library fragments underwent next-generation sequencing using NextSeq 500/550 High Output v2 Kit in 75 cycles (cat. no. FC-404-2005, Illumina, San Diego, CA, USA) on a NextSeq 500 Sequencing System (Illumina, San Diego, CA, USA). Raw fasta reads were quality-checked with FastQC (v0.11.5, Illumina, San Diego, CA, USA) and LiveKraken (v15-065, Illumina, San Diego, CA, USA). 3´end adapters were trimmed with Cutadapt (v1.15, Illumina, San Diego, CA, USA). The trimmed sequences were size filtered for expected miRNA sizes (19 25 bp) and piRNA sizes (24–32 bp) and low-quality ends (Phred < 10) were removed with Cutadapt (v1.15). Statistics from all the preprocessing steps were summarized with MultiQC (v1.4, Illumina, San Diego, CA, USA). Contaminants such as rRNA, tRNA, snoRNA, snRNA, and YRNA were removed from preprocessed reads by mapping (end-to-end) the reads to their sequences with Bowtie (v1.2.1.1, Illumina, San Diego, CA, USA) with a maximum of one mismatch. The preprocessed and cleaned reads were mapped to human miRNA and piRNA sequences downloaded from the miRBase and piRBase database (v1.0, http://www.mirbase.org/ (accessed on 27 September 2022), http://www.regulatoryrna.org/database/piRNA/download.html (accessed on 27 September 2022)) using Bowtie (v1.2.1.1, Illumina, San Diego, CA, USA) with a maximum of two mismatches. Raw Bowtie output was converted to the SAM format using an in-house Perl script and further processed with Samtools (v1.6, Illumina, San Diego, CA, USA), Picard (2.8.2, Illumina, San Diego, CA, USA), and Cgat (v0.3.2, Illumina, San Diego, CA, USA). Feature Counts (v1.5.0, Illumina, San Diego, CA, USA) were used to summarize the miRNA and piRNA counts (minimal overlap of 19 bp).

### 2.3. BioMAI Pipeline Predictor Model

**Figure 3:**
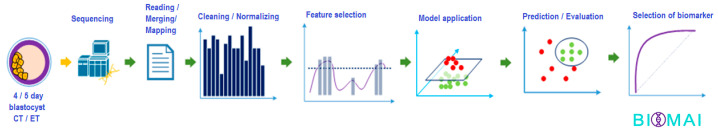
Workflow of creation and validation of embryo quality development using sncRNA from SBM.

The multi-mapped reads were equally divided into the mapped references as fractions. The acquired results were obtained from the sequencing of sncRNAs and the combination of bioinformatics analysis. The BioMAI predictor and a novel machine learning-based tool were developed for predicting human embryonic quality from a spent blastocyst medium. The BioMAI predictor combines all the tasks needed to classify human embryonic quality into one pipeline (Figure 3). Four different models (XG Boost model, Lasso model, Extra Random trees model, and ANOVA coefficient of importance) were used, each for a different function. Specifically, the “must have” bioinformatics tasks such as input quality control, gene mapping, and counting and “required” bioinformatics tasks.

First of all, the number of readings of each sample had to be normalized. Numerous experiments have been performed with complex normalization techniques, such as a reimplementation of TMM (Trimmed Mean of M-values) with a slight enhancement, to assume recognize the proper one, especially for batch correction.

In this study, the following algorithms were trained: (1). the Decision Tree Random Forest model with permutation, (2). the second is XGBoost with GPU acceleration, and (3). the voting classifier. These models were used for some multi-collinearity between expected features, otherwise, Lasso or wGLM (weighted generalized linear model) was used.

The feature selection of the selected molecules significant for the target with one of two states, failure or successful implementation of the embryo, were identified. There were underlying tasks such as pre-filtering features with zero read counts or low variance between groups. Additionally, there was normality testing for the decision of using the proper technique. In this task within the BioMAI predictor, ensemble learning was used (two independent groups of models) that have been implemented with hard voting logic.

The voting technique from different models gave stronger assumptions for selecting significant features. The features that were selected in both above-mentioned models were picked for the next task. The projection of the dataset with the selected features in the previous step gave a useful view of how the two groups are separated.

The next task in the pipeline within the BioMAI predictor was a classification that led to the prediction for evaluated samples. In this task, models with complex hyper-parameters were trained. In this model, we used n-times for predicting each sample in the dataset (“The leave one out method”). Model hyper-parameters were dependent due to some characteristics of the data. The output from this task was a table of predictions. Precisions of predictions were measured with the standard method MSE, ROC, or Precision-recall curve and with the more complex method.

Finally, the BioMAI predictor, for a complex understanding of predictions, especially in the case of an incorrect prediction, was used for the detection of anomalous samples within the fifth model in a row (provided only with training data) and was used in the unsupervised ML method, where the model doesn’t know about depending on variables and knows only independent variables. For this task, the hierarchical clustering model was used.

By using the parameter (fold change ≥ 1 or −1, *p* = 0.05) was identified deregulated piRNA and miRNA molecules were. The coefficient with a *p*-value alpha set to 0.05 was calculated from the number of important molecules with an unknown number of sncRNA molecules significant predictors of the quality embryo from SBM. Based on specific data analysis (explanatory data analysis) using machine learning methods with ensemble learning where we use Support Vector Machine models, Random Forest Classifier, and SGD classifier used in BIOMAI predictor, several predictive miRNA and piRNA biomarkers for embryo quality differentiation in the IVF process were selected.

To determine the accuracy of the final models, the following indicators were used: area under curve ROC curve (AUC ROC), F1 score, and multiple verifications of accuracy with the exchange of input data.

## 3. Results

### 3.1. Ensemble Learning

**Figure 4:**
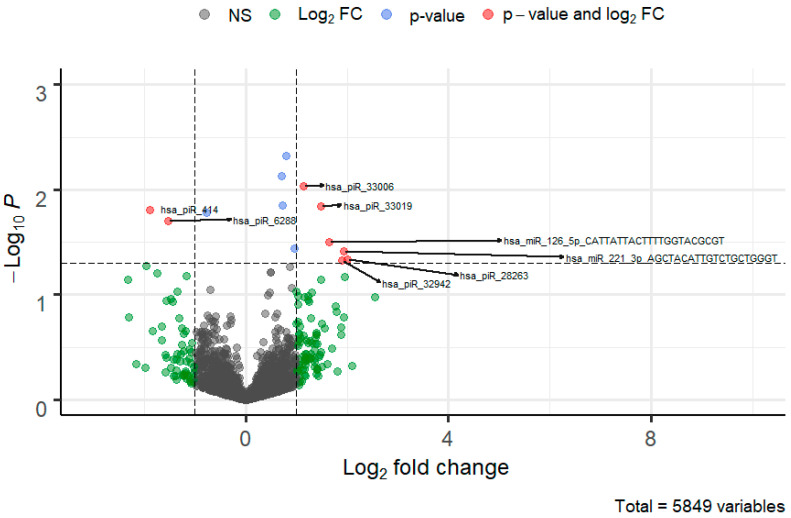
Volcano plot of piRNA and miRNA molecules deregulated in SBM of the quality/competitive embryo in the IVF process. The FC cutoff is set to 1 with a standard *p*-value threshold > 0.05 which visualizes genes that can be potentially differentially expressed.

The analyzed profile of sncRNAs was based on a dataset of SBM samples and obtained 118,338 molecules identified using a publicly available database of sncRNAs (miRBase and piRBase database). The predictor identified deregulated piRNA and miRNA molecules that were differentially expressed in the SBM of the competitive/high-quality embryo group within the IVF process as visualized by a volcano plot (Figure 4). In the beginning, the model started with more than 1000 molecules. After the selection process, the model worked with seven final molecules. The standard DGE analysis with Deseq2 was provided. The result of this analysis did not give any significant difference between the analyzed genes. So far, even a binomial generalized linear model of Deseq2 was unaware. Some setting that was not provided may affect the selection of deferentially expressed genes as normalization type or shrinkage. Additionally, this model is using another method for selection and prediction.

**Figure 5:**
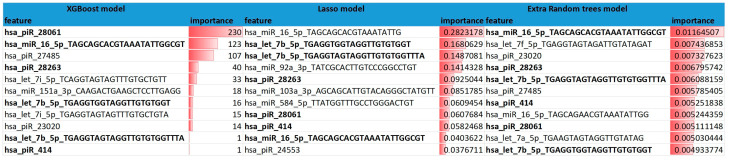
Three decision models using BioMAI predictor and their top 11 features.

The trained decision model evaluated the outputs of the top features as input for the voting algorithm (Figure 5). From this set of the top features sorted by importance value and on recurring significant molecules in the three models (XG Boost model, Lasso model, and Extra Random trees model) and log2 fold change were finally chosen for the voting procession.

The predictor’s voting mechanism for gene selection used three models as a pre-classification, top 15 genes from all three models (which may not be the same in each used model) were statistically compared with ANOVA one-way F-test. The study specifically identified two miRNAs (miR-16-5p, -92a-3p) and five piRNAs (piR-28263, -18682, -23020, -414, -27485) that prognostically and predictively distinguished a high-quality embryo suitable for IVF transmission from a low-quality embryo with 95% sensitivity and 100% specificity with an average accuracy of 86% over mentioned different models. The importance of the obtained biomarker alignment in predicting embryo quality was evaluated after permutations by F-value ANOVA coefficient of importance, *p*-value (Table 1).

### 3.2. Voting Technique

In the exploratory phase of the voting technique, the model used 60 experimental SBM samples of embryo cultivation and three SBM samples as the background of the medium.

The comparison of the PCA projection before and after selecting features visualizes the separation between groups of failure of an implanted embryo (purple) and success of an implanted embryo (blue) and confirms that features were properly selected.

**Figure 6:**
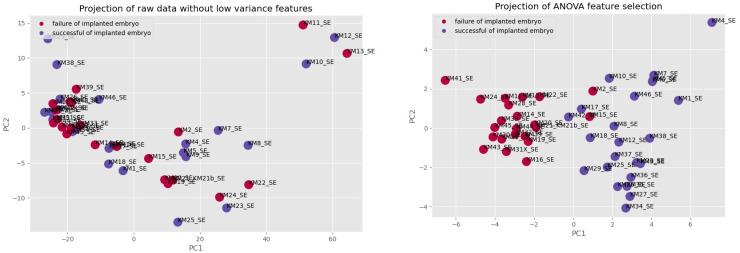
The comparison of PCA projection before and after selected features.

As shown in Figure 6, some SBM samples were predicted to succeed in the IVF process (red dots) and conversely, some samples were predicted to fail the IVF process (blue dots). In some cases, the actual result of the IVF process was contrary to what was predicted by BioMAI. This predicted error was at the level of up to 10% out of all the predicted SBM samples (four samples from 48 samples).

**Figure 7:**
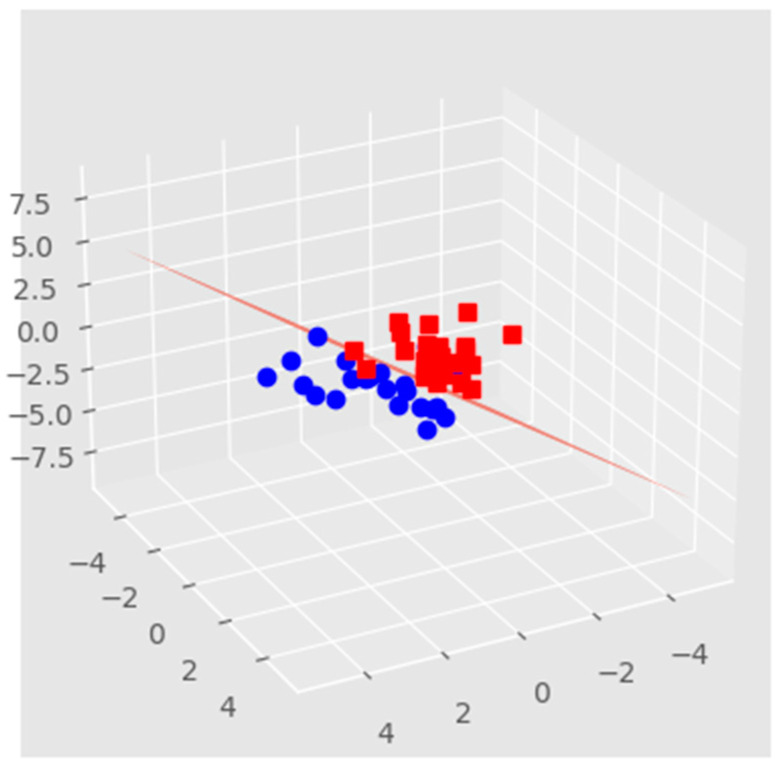
The decision boundary model relates to the previous comparison of PCA projection after BioMAI selection. Red squares represent IVF failure, blue dots represent IVF success.

Model decision boundary analysis, as shown in Figure 7, uses a 3-D principal component (PCA) projection using the plotted model decision boundary plane in the BioMAI predictor. This model sorts the results into two groups: the first (red cubic under plane) that predicts non-competitive, low-quality embryos with the failure of the implanted embryo in the IVF process, and the second (blue cubic over plane) that predicts competitive, quality embryos with the success of the implanted embryo in the IVF process.

**Figure 8:**
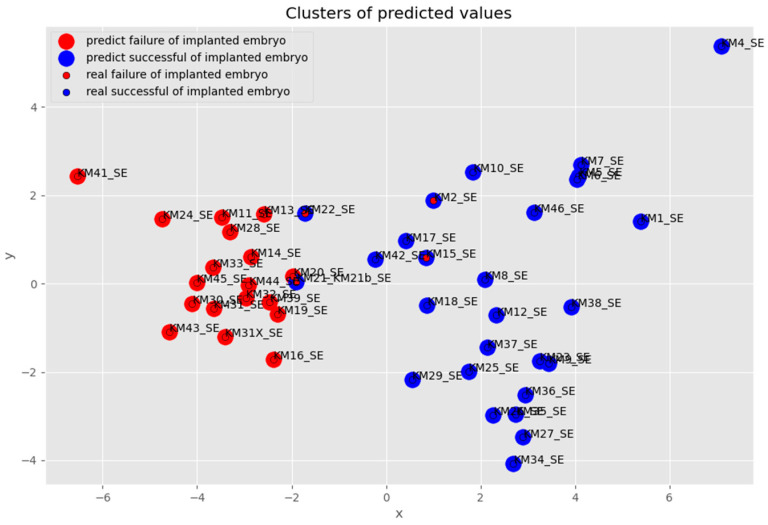
Visualization of identified anomalous samples in successful embryo prediction.

**Figure 9:**
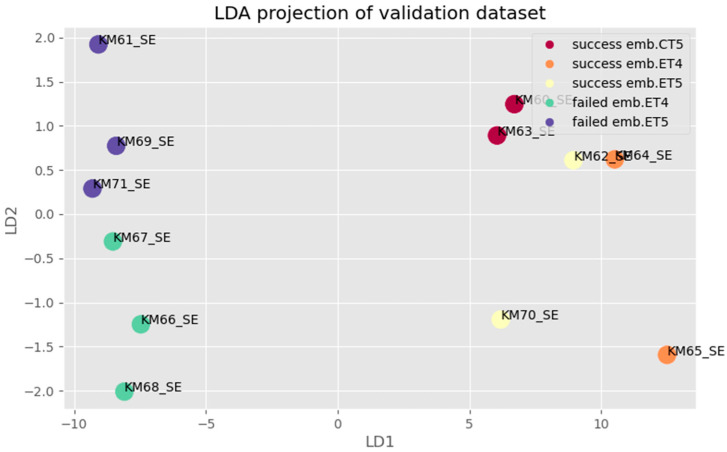
LDA projection for multifactor analysis of validation dataset: Samples with success/failure implanted embryos are grouped of different separation success embryo transfer/ competence embryo and failed embryo transfer/non-competence embryo (legend of abr. ET—fresh embryo transfer, CT—cryo transfer).

Figure 8 shows samples clustered into groups that predict the failure of an implanted embryo (red) and predict the success of an implanted embryo (blue). In all, four samples were misclassified (two of them are not sufficiently legibly displayed in Figure 9—blue balls under the plane) which is up to 10%, and after the elimination of misclassified samples, the accuracy of all predictions was between 86–95%. The analyzed samples of the process were clustered into two groups of the predicted successful and unsuccessful IVF processes. In these two groups, samples KM22_SE, KM21_SE, KM2_SE, and KM15_SE were predicted to be competitive/quality embryos with success in the IVF process, but in reality, they were IVF failures. By deeper analysis, it is seen that samples KM22_SE and KM21_SE are associated with samples predicted, as well as in reality, non-successful in the IVF process. This result suggests the probability of the existence of another reason why these samples were inappropriately predicted.

A blinded step validation (Figure 9) of the BioMAI predictor was also performed by using 12 newly sequenced SBM samples, the samples not used in the exploratory phase on which the model was trained. Based on a specific combination of models of sncRNAs, the BioMAI predictor was able to differentiate a quality/competitive embryo from a non-competitive embryo one with a probability of 100% (all samples were properly predicted).

**Figure 10:**
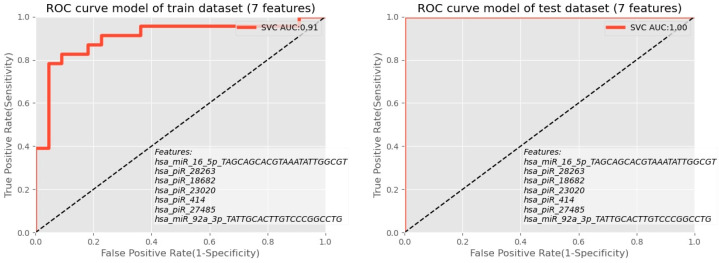
ROC analysis showed the combination of piRNA molecules for the exploratory phase as well as for the validation phase.

Based on the model results and the relation between the accuracy of each model and the number of traits for both monitored indicators of AUC, ROC (Figure 10), and F1 score versus several miRNAs and piRNAs predictive biomarkers, there was agreement that confirmed the number of predictive molecules to be seven molecules and their predictive ability to differentiate embryo quality in the IVF process to be over 86%.

## 4. Discussion

Machine learning algorithms are becoming a revolutionary new milestone in assisted reproduction techniques. Herein the article proposed a novel machine-learning predictor pipeline, BioMAI, combining bioinformatics and biostatistics analyses for predicting high-quality embryos for transfer in the IVF process by using sncRNA from the spent blastocyst media.

**Figure 11:**
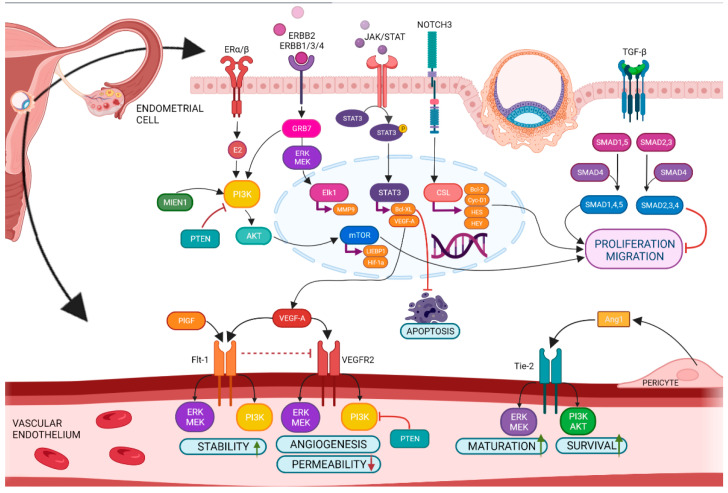
Angiogenesis in the endometrium under physiological conditions. BioMAI-selected miRNAs and piRNAs are directly involved in the regulation of this process.

Among the most important signaling pathways of the implantation of the embryo are VEGF (vascular endothelial growth factor), ERKs (extracellular signal-regulated kinases), MAPKs (Mitogen-activated protein kinases), PI3K/AKT/mTOR (phosphoinositide 3-kinase/protein kinase B/mammalian target of rapamycin) [28], TGF-β (tumor growth factor-beta), Notch (Neurogenic locus notch homolog protein) signaling [29], and ERBB2 (Erb-B2 Receptor Tyrosine Kinase 2) [9]. The family of VEGF (VEGF-A-F, PlGF—placental growth factor, EG-VEGF—endocrine gland-derived vascular endothelial growth factor), and its antagonist sFlt-1 (Soluble Fms-Like Tyrosine Kinase-1) [10], Ang-1/2 (angiopoietin1/2) [11], and endoglin [12] are pivotal for regulation of angiogenesis of mother’s endometrium as well as of embryo (Figure 11). In recent years, differences in miRNA occurrence in SBM of implanted and non-implanted blastocysts have been confirmed (Supplementary Table S1). These miRNAs are relevant in the angiogenesis pathway and represent communication between the blastocyst and uterus endometrium.

Designing accurate biomarkers for embryo selection and detection of its compatibility with the mother’s endometrium is the main challenge for reproductive medicine. The endometrium becomes a well-vascularized tissue with increased vascular permeability, edema, angiogenesis, and perception of blastocyst implantation for a short period (4–5 days) during the middle secretory phase (6–9 days after the peak of luteinizing hormone) of the menstrual cycle [11,30]. The blastocyst (compatible competitive embryo) can invade into uterus endometrium only during the WOI [7]. Perfect timing increases the chance of a successful IVF process. The biggest causes of the negative outcome of the IVF process are poor embryo quality and low endometrial receptivity [31]. One of the most relevant aspects of assisted reproductive technology is the selection of the optimal embryo for transfer. The most widely used method of selecting a suitable embryo for transfer is a standardized evaluation system for selection by an embryologist (Gardner´s scoring system), which means that the evaluation of the embryo’s morphological quality is still burdened by the subjective view of the embryologist [24,32], and an EmbryoScope, which monitor the dynamics of division, compaction, and cavitation with a camera capture to shoot a photograph of every embryo several times an hour in the incubator [33]. The more morphologically appropriate embryo is transferred to the uterus, the higher its probability to implant in the uterus and the higher the success of IVF [34]. However, the implantation capacity of a morphologically high-quality embryo reaches only 50% [35] and the success rate of IVF may therefore still not exceed 30% [7].

Increasing the possibility of the pregnancy after IVF process is also possible by the preimplantation genetic diagnostic which is performed by collecting several cells of the trophectoderm in the stage of the expanded blastocyst and is an invasive intervention to the embryo and can negatively affect its further development [36]. Therefore, emphasis is being placed on the introduction of embryo quality assessment methods that are not subjective, non-invasive, and that are reproducible.

Spent blastocyst medium has shown to be an easily harvested biological material that can be obtained in a non-invasive way for laboratory analyses [15]. Currently, the idea of using artificial intelligence prevails, not only from the point of view of embryo quality detection within the evaluation of images in the EmbryoScope but also as another supporting bioinformatics tool in the detection of embryo quality based on the evaluation of sequencing data, spent blastocyst medium after sequencing [26]. Artificial intelligence could improve the success rate of the IVF process with quick and accurate evaluation of target biomarkers to facilitate clinicians’ decisions.

Currently, artificial intelligence is used in EmbryoScopes, which continuously monitors the development of the embryo for the first five days and, based on an objective assessment of the morphology of the embryo, assesses the potential for the embryo’s viability and its suitability for transfer [33]. Machine learning-based prediction models have been implemented in the field of assisted reproduction to determine a competent embryo for optimizing clinical outcomes by analyzing either the hormonal profile, endometrial thickness, BMI of the patients, etc. [27,37,38] or by characterizing human preimplantation development and/or embryo morphology [39,40,41], but none of them have been concerned with the sncRNA profile from SBM so far.

The major sncRNAs represented in oocytes and early stages of the embryo are miRNAs, piRNAs, and endo-siRNAs (endogenous small interfering RNAs) [42]. The strength of the prediction of embryo implementation into the uterus is narrowly determined by a combination of biomarkers (miRNA and piRNA). MicroRNAs are a family of small non-coding RNAs (19–23 nucleotides) with an important regulatory role in biological processes such as proliferation, differentiation, angiogenesis, migration, apoptosis, and carcinogenesis [43]. The gene silencing mechanism of miRNA is provided by direct binding to specific target mRNA sequences, thus allowing their degradation or translational repression [44]. Piwi-interacting RNAs form the largest class of non-coding RNAs that are slightly longer (24–31 nucleotides) than miRNAs and play a role in silencing transposons to ensure normal gametogenesis and reproduction [45]. The molecules themselves do not have significant predictive power but as a complex form a sensitive predictive biomarker. The sncRNAs are important as biomarkers either in combination with protein expressions of affected regulatory points of the angiogenic pathway (such as PI3K, ERK, JAK/STAT, NOTCH3, TGF-β/ALK1, and TGF-β/ALK5) [9,10,11,12,28,29] or as a cluster of sncRNAs that affects “jointly” the change in the expression of targets but nowhere sncRNA by itself [23,43,44,45].

The BioMAI predictor identified two miRNAs, namely miR-92a-3p, and miR-16-5p, and five piRNAs, namely piR-28263, -18682, -23020, -414, and -27485, as potential biomarkers for the identification of embryos suitable for implantation, from an 11 sncRNAs with the high importance score based on the combination of three selective models (XGBoost, Lasso, Extra Random trees) from 118,338 molecules identified in gene sequencing.

The BioMAI-predicted miR-16-5p was previously described as an age-related downregulated molecule in follicular fluid [46] and was also upregulated in patients with PCOS (polycystic ovary syndrome) during pregnancy [47]. Another study found miR-16-5p in the SBM of both implanted and non-implanted blastocysts, however, a higher level was found in the SBM of successfully implanted blastocysts [18]. MiR-16-5p was observed downregulated in case of fetal growth restriction during pregnancy in the mother´s peripheral blood [47] and it is a putative marker of fetal growth restriction. Further, miR-16-5p was also studied as a major molecule of interest in embryo-conditioned culture media by Russell et al. [48]. The role of this miRNA in gestational diabetes mellitus [49] and a link to diabetic embryopathy, especially neural and cardiac malformations, were studied in a mice model. The inflammatory diseases in the first trimester of pregnancy were associated with the dysregulation of miR-16 [50]. This could lead to pregnancy failure by proxy, as it has been observed, that disrupted inflammatory responses to infectious diseases in the first trimester such as those of the urinary tract can lead to miscarriage in early pregnancy or preeclampsia in later pregnancy [51,52,53].

The second predicted miRNA by the BioMAI predicting system was miR-92a-3p which is associated with a higher success of blastocyst implantation [20,54]. The level of miR-92a-3p was also studied in the cases of endometriosis and PCOS [55,56] where the level was downregulated. Endometriosis, as well as PCOS, is a well-known reason for infertility that reduces the implantation rate after embryo transfer [57,58].

The other group of sncRNAs, piRNAs, have the guiding role in heterochromatin structure formation during embryogenesis [59]. The silent chromatin state is apparently transmitted by the heterochromatin system during development when the piRNA system is largely absent in nongonadal somatic cells [59]. PIWI proteins expressed in the germline affect the reproductive system in both females and males. For example, in Drosophila and zebrafish, impairment of the PIWI role leads to sterility, disruption in the piwi genes leads to sterility exclusively in males, and impairment of the AGO3 protein in Drosophila leads to sterility in females and semi-sterility in males [42].

In human oocytes, long piRNAs, miRNAs, and os-piRNAs (oocyte short piRNAs) are expressed and lack endo-siRNAs [42]. It should be noted that many oocytes piRNA have been derived from poorly annotated genomic regions, which may explain that os-piRNAs have other roles such as general regulation of gene expression during oocyte development in addition to TEs (transposable elements) silencing. The cleavage of expressed TE transcripts during secondary piRNA biogenesis reflects what is probably the primary and most highly conserved function of the PIWI pathway. The same goes for the post-transcriptional gene silencing (PTGS) of TEs through the slicer activity of piRISCs and transcriptional gene silencing (TGS), primarily through directing DNA methylation and histone modifications [60].

Just a few articles that focus on BioMAI-predicted piwi-interacting RNAs and their role in fertility were published at the time of writing this article. Piwi-interacting RNA molecules and PIWI proteins have an important function in the development of germ cells and are directly connected to fertility in both genders [61]. Recent studies have shown different expression levels in terms of the morphological grade of the embryo and the implantation potential of the piRNA molecules from SBM, namely piR20401, piR16735, piR19675, piR20326, piR17716 [17].

Females with the defective piRNA’s biogenesis including the PIWI proteins family, exhibit normal oogenesis and fertility [62]. The reciprocal regulation of ncRNAs in embryonic development linked with the piRNAs with sequences homologous to the 5′ seed region of miR-17-5p/3p was studied in a mice model by Du et al. [63]. The role of piRNAs in maternal mRNA stability in oocytes and embryos studied in the golden hamster model [64] showed piRNA pathways in the regulation of female fertility. Another review showed piRNA’s function in oocytes and embryos [65]. The predominant class of small RNAs is piRNAs in Xenopus eggs and oocytes (an important model for understanding piRNA biogenesis, which is more accurate than the mice model) [66] where they are markers of epigenetic inheritance. However, there is much more evidence on the role of piRNA in male fertility (level in testicles and ejaculation) [66,67,68,69].

Herein the article proposed a pipeline predictor, BioMAI, by using artificial intelligence and combining bioinformatics and biostatistics analyses for predicting high-quality embryos for transfer in the IVF process by using sncRNA from the spent blastocyst media.

## 5. Conclusions

Besides gene expression analysis of target datasets, executed a multifactor analysis of the validation dataset was provided. The hypothesis tested, whether the pipeline is able to distinguish between cultivation media of embryos of different ages (4th and 5th day of cultivation) as well as previous storage (fresh or cryo-preserved). It was managed to provide deeper and more detailed differentiation of competent and incompetent embryos based on a subset of molecules miR-92a-3p, -16-5p, piR-28263, -18682, -23020, -414, -27485 available from the spent cultivation medium of the embryo.

The development of a successful embryo score prediction tool with a combination of presence tools used for precise identification and selection of competitive embryos by EmbryoScope and sncRNAs from the BioMAI model forms a predictive model enabling the prognosis of the competence of the embryo for transfer to the uterus during the IVF process, considering the conditions of cultivation and storage of the embryos. This predictor could help both patients and medical practitioners make a personalized decision using the tool to distinguish competitive from non-competitive embryos and increase the success of embryo implantation. This study used artificial intelligence to identify specific two miRNAs and five piRNAs from SBM’s sequencing data, which predictively select a competitive quality embryo suitable for the IVF transfer to the uterus from a non-competitive, low-quality embryo, with 86% accuracy.

Verification of the BioMAI prediction model in a clinical study could support the use of BioMAI as an auxiliary tool for identifying a competent embryo. This could subsequently help increase the success of the IVF process, reducing repeated transfers in women and reducing the economic costs of infertility treatment, as well as improving the mental health of infertile couples.

## 6. Patents

WO 2021/177904 A4: “Non-invasive successfulness test of in vitro fertilization process ”, TRL 5, C12Q 1/6883.

## Figures and Tables

**Table 1:** Piwi-interacting RNA and micro RNA molecules are deregulated in the SBM of the embryo in the IVF process.

	F-Value ANOVA Coefficient of Importance	*p*-Value
hsa_miR_16_5p	8,853,048	0.004786
hsa_piR_28263	6,154,501	0.017098
hsa_piR_18682	5,506,564	0.023622
hsa_piR_23020	5,341,872	0.025678
hsa_piR_414	5,103,818	0.028998
hsa_piR_27485	5,028,244	0.030147
hsa_miR_92a_3p	499,102	0.030731

## Data Availability

http://www.mirbase.org/ (accessed on 27 September 2022); http://www.regulatoryrna.org/database/piRNA/download.html (accessed on 27 September 2022).

## Supplementary Materials

The following supporting information can be downloaded at: https://www.mdpi.com/article/10.3390/biom12111687/s1. Table S1. Selected miRNAs from the SBM and their respective target genes. References [9,32,33,34,35,36,37,38,39,40] are cited in the supplementary materials.
