# Supplementary material for: Small Non-Coding RNAs as New Biomarkers to Evaluate the Quality of the Embryo in the IVF Process
Source: Biomolecules. 2022 Nov 14;12(11):1687. doi: 10.3390/biom12111687 (PMC9687427; doi:10.3390/biom12111687)
Supplement: Supplementary file 1 [file biomolecules-12-01687-s001.zip › biomolecules-1953351-supplementary.pdf]

biomolecules

MDPI

Article

Small non-coding RNAs as new biomarkers to evaluate the quality of the embryo in the IVF process

Silvia Toporcerová[1,2†], Ivana Špaková[3†], Katarína Šoltys[4], Zuzana Klepcová[3], Marek Kľoc[5], Júlia Bohošová[6], Karolína Trachtová[6], Lucia Peterová[2], Helena Mičková[7], Peter Urdzík[1], Mária Mareková[3], Ondřej Slabý[6], Miroslava Rabajdová[3,5*]

1 Department of Gynaecology and Obstetrics, Faculty of Medicine, Pavol Jozef Šafárik University in Košice, Košice, Slovakia
2 Gyncare a.s., Košice, Slovakia
3 Department of Medical and Clinical Biochemistry, Faculty of Medicine, Pavol Jozef Šafárik University in Košice, Košice, Slovakia
4 Faculty of Natural Sciences, Comenius University in Bratislava, Bratislava, Slovakia
5 SAFTRA-BioMAI, Pavol Jozef Šafárik University in Košice, Košice, Slovakia
6 Central European Institute of Technology, Masaryk University, Brno, Czech Republic
7 Department of Medical Biology, Faculty of Medicine, Pavol Jozef Šafárik University in Košice, Košice, Slovakia
† both authors contributing as a first author
* Correspondence: miroslava.rabajdova@upjs.sk; Tel.: +421 55 234 3377

Citation: Lastname, F.; Lastname, F.; Lastname, F. Title. *Biomolecules* **2022**, *12*, x. https://doi.org/10.3390/xxxxx

Academic Editor: Firstname Lastname

Received: date
Accepted: date
Published: date

Publisher's Note: MDPI stays neutral with regard to jurisdictional claims in published maps and institutional affiliations.

Copyright: © 2022 by the authors. Submitted for possible open access publication under the terms and conditions of the Creative Commons Attribution (CC BY) license (https://creativecommons.org/licenses/by/4.0/).

Table 1. Selected miRNAs from the SBM and their respective target genes.

process	miRNA with successfully implanted blastocysts	target genes	miRNA with non-successfully implanted blastocysts	target genes
proliferation	miR-20a [9]	PTEN, ATG7, TIMP2	miR-373-3p [33]	----
	miR-30c [9]	APC, KRAS, PIK3CD	miR-518a-3p [33]	NIK
	miR-19b-3p [33]	PTEN	miR-191 [34]	EGR1
	miR-372-3p [33]	----		
	miR-634 [37]	mTOR		
	let-7b-5p [32, 38]	TGFBR1, NRAS		
angiogenesis	miR-29c-3p [37]	COL4	miR-373-3p [33]	VEGF-A
	miR-20a [39]	VEGF-A, HIF-1α		
	miR-634 [37]	JAG		
viability	miR-20a [40]	BCL2	miR-182-5p [33]	CASP9
	miR-92a-3p [38]	-----		
	let-7b-5p [38]	NRAS		
cell cycle	miR-20a [9]	CDKN1A/p21, CCND1	miR-142-3p [36]	CCND1
			miR-372 [34]	CDK2, CDK6, MAPK3, CYCLIN A1
			miR-302a-3p [33]	HMGA2, CCND1, OCT4, SOX2
			miR-191 [34]	CDK6, MAPK3
apoptosis	miR-634 [37]	mTOR	miR-372 [34]	MAP3K1, CDK6
	let-7b-5p [32]	TNFRSF10B	miR-518a-3p [33]	NIK
			miR-302a-3p [33]	HMGA2
			miR-191 [34]	MAP3K, CDK6
implantation	let-7b-5p [32]	IGF1R, MUC1	miR-661 [35]	MDM2
	let-7i-5p [32]	AKT2	miR-519d-3p [33]	MMP2
			miR-302a-3p [33]	SOCS5, HMGA2
blastocyst’s development	let-7b-5p [32]	AKT2, FOXO1, IGF2BP2		
	let-7i-5p [32]	TEAD1, BUB1B, FOXO1		
